# Development and internal validation of a KELIM-integrated prognostic nomogram for long-term survival prediction in epithelial ovarian cancer

**DOI:** 10.3389/fonc.2026.1934729

**Published:** 2026-08-31

**Authors:** Qianqian Liu, Yibin Liu, Guohong Qi, Mengyao Su, Xueqian Miao, Zhiqiang Zhang, Xianghua Huang, Yanfang Du, Zhongkang Li, Li Meng

**Affiliations:** 1 Department of Obstetrics and Gynecology, The Second Hospital of Hebei Medical University, Shijiazhuang, Hebei, China; 2 Hebei Key Laboratory of Regenerative Medicine of Obstetrics and Gynecology, The Second Hospital of Hebei Medical University, Shijiazhuang, Hebei, China; 3 National Clinical Key Specialty Construction Project of Gynecology, Shijiazhuang, Hebei, China; 4 Department of Birth Control, Tianjin Central Hospital of Obstetrics and Gynecology, Tianjin, China

**Keywords:** epithelial ovarian cancer, KELIM, overall survival, prognosis, prognostic nomogram

## Abstract

**Introduction:**

Epithelial ovarian cancer (EOC) remains the most lethal gynecological malignancy, and accurate prediction of long-term survival remains challenging. This study aimed to evaluate the prognostic associations of prespecified clinically relevant factors with overall survival (OS) and to develop and internally validate a KELIM-integrated prognostic nomogram.

**Methods:**

A total of 100 patients with EOC treated at the Second Hospital of Hebei Medical University between May 2014 and June 2021 were retrospectively analyzed. Five clinically relevant predictors were prespecified for multivariable modeling: age, histological subtype, FIGO stage, postoperative residual disease, and KELIM. The primary multivariable analysis included 92 patients with complete data for all five predictors. Internal validation was performed using 1,000 bootstrap resamples.

**Results:**

During a median follow-up of 87 months (95% CI, 65–91 months), 60 deaths occurred, and the Kaplan–Meier-estimated median OS was 57 months (95% CI, 44–70 months). Increasing age, advanced FIGO stage, KELIM <1, and gross postoperative residual disease were independently associated with poorer OS, whereas histological subtype was not independently associated with OS. The optimism-corrected C-index was 0.779. The optimism-corrected time-dependent AUCs for predicting 1-, 3-, and 5-year OS were 0.917, 0.865, and 0.799, respectively, compared with 0.699, 0.733, and 0.679 for FIGO stage alone.

**Discussion:**

Integrating KELIM with established clinical and surgical prognostic factors may enhance individualized survival prediction and risk stratification in patients with EOC. External validation in independent cohorts is required before clinical implementation.

## 1 Introduction

Epithelial ovarian cancer (EOC) remains the most lethal gynecological malignancy and continues to represent a major global health challenge (1, 2). Although advances have been made in cytoreductive surgery, platinum-based chemotherapy and maintenance therapy, a substantial proportion of patients are diagnosed at an advanced stage, when extensive peritoneal dissemination has already occurred (2). In China, the disease burden of ovarian cancer has also increased over recent decades, partly reflecting population aging and the lack of effective early screening strategies (3). Therefore, improving prognostic assessment is essential for individualized risk stratification and long-term management of patients with EOC.

Survival outcomes in EOC are highly heterogeneous and are influenced by both tumor burden at diagnosis and response to treatment. FIGO stage remains the cornerstone of clinical assessment, as it reflects the anatomical extent of disease and guides initial treatment planning (4). Histological subtype is also clinically relevant, with high-grade serous ovarian cancer accounting for the majority of cases and generally showing aggressive biological behavior (5). Among surgical factors, postoperative residual disease is one of the most consistently validated prognostic indicators. Complete macroscopic cytoreduction with no gross residual disease (R0) is associated with superior survival compared with any gross postoperative residual disease (6). However, these conventional clinicopathological factors are largely static and cannot fully capture the biological behavior of the tumor or its intrinsic chemosensitivity.

Serum CA125 is widely used in EOC for diagnosis, treatment monitoring and surveillance (7). Nevertheless, a single CA125 value has limited prognostic value because it may be influenced by tumor burden and non-malignant conditions such as endometriosis, menstruation and peritoneal inflammation (7). Compared with static CA125 measurements, dynamic changes in CA125 during chemotherapy may provide more clinically meaningful information regarding early treatment response. The modeled CA125 elimination rate constant K, commonly referred to as KELIM, is calculated from serial CA125 measurements during the early phase of platinum-based chemotherapy and has emerged as a marker of tumor chemosensitivity (8). Previous studies, including the CHIVA study, the CALYPSO trial and the GOG-0218 validation study, have demonstrated that KELIM is associated with chemotherapy response, progression-free survival and overall survival in ovarian cancer (8–10). These findings suggest that KELIM may provide prognostic information beyond conventional staging and postoperative residual disease.

In parallel with the development of dynamic biomarkers, prognostic models have increasingly been used to integrate multiple clinical variables for individualized survival prediction. Several nomograms for ovarian cancer have incorporated factors such as age, stage, histological subtype, CA125 level and treatment-related variables (11, 12). However, these models predominantly rely on baseline clinicopathological characteristics and do not include dynamic treatment-response indicators. Therefore, a prognostic model incorporating both conventional prognostic factors and CA125 kinetic information may provide a more comprehensive approach to survival prediction in EOC.

In the present study, we retrospectively analyzed 100 patients with EOC treated at the Second Hospital of Hebei Medical University between May 2014 and June 2021. The aims were to evaluate the prognostic associations of prespecified clinically relevant factors with OS and to develop and internally validate a KELIM-integrated prognostic nomogram, including age, histological subtype, FIGO stage, postoperative residual disease and KELIM. By integrating patient characteristics, tumor histology, anatomical disease extent, surgical outcome and early CA125 kinetics, this study sought to develop a practical prognostic tool based on routinely available clinical data for individualized long-term survival prediction in patients with EOC.

## 2 Materials and methods

### 2.1 Patients and study design

The present retrospective cohort study was conducted at the Second Hospital of Hebei Medical University, a tertiary referral center in northern China. The study aimed to evaluate the prognostic associations of prespecified clinically relevant factors with OS and to develop and internally validate a KELIM-integrated prognostic nomogram. Patients with EOC treated between May 2014 and June 2021 were screened.

The inclusion criteria were as follows: i) Histopathologically confirmed primary EOC according to the World Health Organization classification; ii) initial treatment performed at the Second Hospital of Hebei Medical University; iii) receipt of primary comprehensive staging surgery or cytoreductive surgery followed by platinum-based chemotherapy; iv) available preoperative and serial peri-chemotherapy CA125 measurements required for KELIM calculation; v) available core clinical, pathological, treatment and follow-up information required to establish study eligibility and ascertain OS; and vi) age ≥18 years. The exclusion criteria were as follows: i) Borderline ovarian tumors or non-epithelial ovarian tumors, including germ cell tumors and sex cord-stromal tumors; ii) recurrent disease at initial presentation; iii) previous treatment for other malignancies before EOC diagnosis; iv) preoperative chemotherapy or other neoadjuvant treatment; and v) insufficient baseline information to establish study eligibility or ascertain the primary outcome, or loss to follow-up. A total of 100 eligible patients were included in the retrospective cohort. Among them, 92 patients had complete data for all five prespecified predictors and constituted the primary model-development cohort.

### 2.2 Data collection

Clinical, surgical, pathological and laboratory data were retrospectively collected from the electronic medical record system. Clinical variables included age at diagnosis and body mass index. Surgical and disease-related variables included ascites volume, malignant cells in ascites, omental metastasis, liver metastasis and postoperative residual disease. Postoperative treatment variables included the interval from surgery to chemotherapy initiation and the number of chemotherapy cycles completed. Pathological variables included FIGO stage and histological subtype. Laboratory variables included preoperative CA125 levels and serial CA125 measurements obtained during platinum-based chemotherapy.

Postoperative residual disease was reassessed from the operative records and initially classified as R0 (no gross residual disease), R1 (gross residual disease ≤1 cm) or R2 (gross residual disease >1 cm). For the primary prognostic analyses, residual disease was categorized as R0 versus any gross residual disease (R1/R2). Residual disease status could be unequivocally determined in 92 patients. In eight patients who underwent staging, restaging, fertility-sparing or non-cytoreductive procedures, the available records did not permit reliable classification as R0, R1 or R2. These patients were retained in analyses not requiring residual disease status but were excluded from complete-case analyses involving this variable.

One patient had the preoperative CA125 level documented qualitatively as within the normal reference range without an exact numerical value. This patient was classified as having CA125 <35 U/ml for categorical analysis but was excluded from numerical summaries requiring an exact CA125 value. In one patient, ascites volume was documented only as >500 ml; this patient was excluded from analyses requiring an exact continuous ascites volume.

### 2.3 KELIM calculation

KELIM was used as a dynamic indicator of early tumor chemosensitivity. It was calculated using a validated nonlinear mixed-effects model based on longitudinal CA125 values obtained during the early phase of platinum-based chemotherapy, as previously described (8). For each patient, at least three CA125 measurements obtained within the first 100 days after chemotherapy initiation were included in the calculation. A publicly available CA125 KELIM calculator was used to generate an individualized KELIM value. For prognostic analyses, KELIM was categorized using the established cutoff value of 1 as KELIM <1 versus KELIM ≥1. A KELIM value ≥1 was considered to indicate favorable CA125 elimination kinetics and higher chemosensitivity, whereas a KELIM value <1 indicated unfavorable CA125 kinetics.

### 2.4 Follow-up and outcome definition

Follow-up data were obtained through outpatient visits and telephone interviews using a standardized follow-up form developed for this study. Patients were followed every 3 months during the first 2 years after treatment and every 6 months thereafter when clinically appropriate. Follow-up assessments included survival status, date of death or last contact, physical examination, imaging evaluation and CA125 measurement.

The primary endpoint was overall survival (OS), defined as the interval from the date of initial surgery to death from any cause or the date of last follow-up. The follow-up cutoff date was October 1, 2024. Patients who were alive at the last follow-up were right-censored.

### 2.5 Ethics approval

The present study was approved by the Research Ethics Committee of the Second Hospital of Hebei Medical University (approval no. 2025-R271). The study was conducted in accordance with the Declaration of Helsinki. Owing to the retrospective design and use of anonymized clinical data, the requirement for written informed consent was waived by the ethics committee. All patient information was de-identified before analysis.

### 2.6 Statistical analysis

All statistical analyses were performed using SPSS version 27.0 (IBM Corp.) and R version 4.4.0 (R Foundation for Statistical Computing). Continuous variables are presented as the median and interquartile range or the mean ± standard deviation, as appropriate, and categorical variables are presented as numbers and percentages.

Overall survival was estimated using the Kaplan-Meier method. Median OS and the 1-, 3- and 5-year OS probabilities were reported with corresponding 95% confidence intervals (CIs). Confidence intervals for Kaplan-Meier survival estimates were calculated using Greenwood variance with log-log transformation. Median follow-up duration was estimated using the reverse Kaplan-Meier method. Survival curves for predefined categorical variables were compared using the log-rank test.

Age and ascites volume were retained as continuous variables in survival analyses to avoid bias associated with outcome-dependent dichotomization. For clinical interpretability, the effect of age was expressed per 10-year increase and the effect of ascites volume per 500-ml increase. BMI was categorized as <24 versus ≥24 kg/m², preoperative CA125 was categorized using the conventional cutoff value of 35 U/ml, the interval from surgery to chemotherapy initiation was modeled per 7-day increase and the number of chemotherapy cycles was categorized as ≤6 versus >6 cycles. KELIM was categorized using the established cutoff value of 1. FIGO stage was grouped as stages I-II versus III-IV for regression modeling because of the limited numbers of patients with stage II and IV disease. Postoperative residual disease was categorized as R0 versus any gross residual disease.

Univariable Cox proportional hazards regression analyses were performed to explore associations between individual clinicopathological variables and OS. To reduce the risk of overfitting given the limited number of outcome events, candidate predictors for multivariable modeling were restricted *a priori* on the basis of established clinical relevance and the primary objective of the study. Five predictors were prespecified: age at diagnosis, histological subtype (HGSOC vs. non-HGSOC), FIGO stage (I-II vs. III-IV), KELIM (<1 vs. ≥1) and postoperative residual disease (R0 vs. any gross residual disease). Selection of variables for the multivariable model was therefore not based on statistical significance in the univariable analyses. The primary modeling cohort comprised 92 complete cases with 59 deaths, corresponding to approximately 11.8 outcome events per prespecified predictor parameter. Hazard ratios (HRs) and 95% CIs were calculated. The proportional hazards assumption was assessed before final model interpretation. A two-sided P<0.05 was considered statistically significant.

No statistical imputation was performed. Analyses involving postoperative residual disease were conducted using complete cases. To assess the potential influence of the eight patients with indeterminate residual disease status, extreme-case sensitivity analyses were performed by alternatively classifying all eight patients as R0 or all eight as having gross residual disease and refitting the prespecified multivariable model.

### 2.7 Nomogram construction and validation

A prognostic nomogram was constructed from the full multivariable Cox model containing all five prespecified predictors: age, histological subtype, FIGO stage, KELIM and postoperative residual disease. All five variables were retained in the prognostic model irrespective of their individual P values. Because complete postoperative residual disease information was available for 92 patients, model development and internal validation were performed in these 92 complete cases. The nomogram was used to estimate individual 1-, 3- and 5-year OS probabilities.

Internal validation was performed using 1,000 bootstrap resamples to quantify model optimism. In each bootstrap sample, the prespecified five-predictor Cox model was refitted, and model performance was evaluated both in the bootstrap sample and in the original development cohort. Optimism was defined as the difference between bootstrap-sample performance and test performance in the original cohort and was averaged across the 1,000 resamples. Optimism-corrected performance was obtained by subtracting the mean optimism from the corresponding apparent performance estimate.

Model discrimination was assessed using Harrell’s concordance index (C-index). Time-dependent receiver operating characteristic analysis was used to estimate AUCs for 1-, 3- and 5-year OS. Apparent and bootstrap optimism-corrected C-index and AUC estimates were calculated for the five-predictor model. The discriminatory performance of the model was additionally compared with that of FIGO stage alone in the same development cohort.

Calibration at 1, 3 and 5 years was evaluated by comparing model-predicted survival probabilities with Kaplan–Meier-estimated observed survival probabilities. Both apparent and bootstrap optimism-corrected calibration estimates were examined. Decision curve analysis was performed to explore the potential clinical net benefit of the model for predicting 1-, 3- and 5-year mortality across a range of threshold probabilities. The development and reporting of the prognostic model were performed with reference to the TRIPOD statement (13).

## 3 Results

### 3.1 Baseline characteristics

A total of 100 patients with pathologically confirmed EOC were included in the study. The median age at diagnosis was 51.0 years (IQR, 45.8-59.3 years). High-grade serous ovarian cancer (HGSOC) was the most common histological subtype and was diagnosed in 54 patients (54.0%), whereas 46 patients (46.0%) had non-HGSOC histological subtypes.

According to FIGO stage, 35 patients (35.0%) had stage I disease, 8 (8.0%) had stage II disease, 54 (54.0%) had stage III disease and 3 (3.0%) had stage IV disease. Thus, 43 patients (43.0%) had early-stage disease (FIGO I-II) and 57 (57.0%) had advanced-stage disease (FIGO III-IV). The median KELIM value was 1.10 (IQR, 0.79-1.60); 41 patients (41.0%) had KELIM <1 and 59 (59.0%) had KELIM ≥1.

Postoperative residual disease status was evaluable in 92 patients. Among these patients, 47 (51.1%) achieved no gross residual disease (R0), whereas 45 (48.9%) had any gross residual disease (R1/R2). In the remaining eight patients, residual disease status could not be reliably classified from the available operative records. Among patients with numerical measurements available, the median preoperative CA125 level was 598.7 U/ml (IQR, 89.5-1771.5 U/ml; n=99), and the median ascites volume was 200 ml (IQR, 20–1600 ml; n=99). Malignant cells in ascites were detected in 57 patients (57.0%), omental metastasis in 46 (46.0%) and liver metastasis in 3 (3.0%). Additional baseline and treatment-related characteristics are summarized in **Table 1**. Exploratory associations between KELIM and selected clinical and clinicopathological characteristics are summarized in **Supplementary Table 1**.

**Table 1:** Baseline clinical and clinicopathological characteristics of patients with epithelial ovarian cancer.

Variable	Total cohort
Total, n	100
Age, years, median (IQR)	51.0 (45.8–59.3)
BMI, kg/m², median (IQR)	24.0 (21.7–26.7)
Histological subtype, n (%)	
HGSOC	54 (54.0)
Non-HGSOC	46 (46.0)
FIGO stage, n (%)	
Stage I	35 (35.0)
Stage II	8 (8.0)
Stage III	54 (54.0)
Stage IV	3 (3.0)
KELIM	
KELIM value, median (IQR)	1.10 (0.79–1.60)
<1, n (%)	41 (41.0)
≥1, n (%)	59 (59.0)
Postoperative residual disease, n=92, n (%)	
R0	47 (51.1)
R1	32 (34.8)
R2	13 (14.1)
Preoperative CA125, U/ml, median (IQR), n=99	598.7 (89.5–1771.5)
Preoperative CA125 <35 U/ml, n (%)	14 (14.0)
Preoperative CA125 ≥35 U/ml, n (%)	86 (86.0)
Ascites volume, ml, median (IQR), n=99	200 (20–1600)
Malignant cells in ascites, n (%)	
Absent	43 (43.0)
Present	57 (57.0)
Omental metastasis, n (%)	
Absent	54 (54.0)
Present	46 (46.0)
Liver metastasis, n (%)	
Absent	97 (97.0)
Present	3 (3.0)
Interval from surgery to chemotherapy initiation, days, median (IQR)	11 (10–14)
Number of chemotherapy cycles, median (IQR)	6 (6–8)
≤6 cycles, n (%)	54 (54.0)
>6 cycles, n (%)	46 (46.0)

Data are presented as n (%) unless otherwise indicated. Continuous variables are presented as median (interquartile range). Postoperative residual disease status was evaluable in 92 patients; R1 and R2 were combined as any gross residual disease for the primary prognostic analyses. One patient had the preoperative CA125 level documented qualitatively as within the normal reference range without an exact numerical value; this patient was included in the categorical CA125 analysis as <35 U/ml but excluded from numerical summaries of CA125. One patient had ascites volume documented only as >500 ml and was excluded from analyses requiring an exact continuous ascites volume. BMI, body mass index; HGSOC, high-grade serous ovarian cancer; FIGO, International Federation of Gynecology and Obstetrics; KELIM, modeled CA125 elimination rate constant K; R0, no gross residual disease.

### 3.2 Survival outcomes and univariable analyses

During follow-up, 60 patients died and 40 were alive at the last follow-up and were censored. The observed follow-up times ranged from 11 to 116 months. The median follow-up duration, estimated using the reverse Kaplan-Meier method, was 87 months (95% CI, 65–91 months). The Kaplan-Meier-estimated median OS was 57 months (95% CI, 44–70 months). The estimated 1-, 3- and 5-year OS rates were 96.0% (95% CI, 89.7-98.5%), 69.0% (95% CI, 58.9-77.1%) and 47.7% (95% CI, 37.2-57.5%), respectively.

In univariable Cox regression analyses, increasing age was associated with poorer OS (per 10-year increase: HR, 1.36; 95% CI, 1.07-1.73; P = 0.013). HGSOC histology (HR, 3.51; 95% CI, 1.95-6.31; P<0.001), advanced FIGO stage (III-IV vs. I-II: HR, 5.62; 95% CI, 2.91-10.86; P<0.001) and any gross postoperative residual disease (vs. R0: HR, 3.75; 95% CI, 2.16-6.50; P<0.001) were also associated with poorer OS. In contrast, KELIM ≥1 was associated with a lower hazard of death (HR, 0.34; 95% CI, 0.20-0.56; P<0.001). Increasing ascites volume, malignant cells in ascites, omental metastasis and a longer interval from surgery to chemotherapy initiation were also associated with OS, whereas BMI, preoperative CA125 category, liver metastasis and chemotherapy cycle category were not statistically significant (**Table 2**).

**Table 2:** Univariable Cox proportional hazards regression analyses for overall survival in patients with epithelial ovarian cancer.

Predictor	n	HR	95% CI	P-value
Age, per 10-year increase	100	1.36	1.07–1.73	0.013
BMI, ≥24 vs. <24 kg/m²	100	1.11	0.67–1.85	0.682
HGSOC vs. non-HGSOC	100	3.51	1.95–6.31	<0.001
FIGO III–IV vs. I–II	100	5.62	2.91–10.86	<0.001
KELIM ≥1 vs. <1	100	0.34	0.20–0.56	<0.001
Any gross residual disease vs. R0	92	3.75	2.16–6.50	<0.001
Preoperative CA125 ≥35 vs. <35 U/ml	100	2.64	0.95–7.28	0.061
Ascites volume, per 500-ml increase	99	1.16	1.07–1.25	<0.001
Malignant cells in ascites, present vs. absent	100	2.22	1.29–3.80	0.004
Omental metastasis, present vs. absent	100	2.96	1.73–5.06	<0.001
Liver metastasis, present vs. absent	100	1.77	0.55–5.68	0.339
Interval from surgery to chemotherapy initiation, per 7-day increase	100	1.25	1.04–1.49	0.018
Chemotherapy cycles, >6 vs. ≤6	100	1.55	0.93–2.58	0.091

HRs >1 indicate a higher hazard of death for increasing continuous predictors or for the first category relative to the reference category, whereas HRs <1 indicate a lower hazard of death. Postoperative residual disease analysis included the 92 patients with evaluable residual disease status. Ascites volume analysis included 99 patients with an exact numerical value available. HR, hazard ratio; CI, confidence interval; BMI, body mass index; HGSOC, high-grade serous ovarian cancer; FIGO, International Federation of Gynecology and Obstetrics; KELIM, modeled CA125 elimination rate constant K; R0, no gross residual disease.

### 3.3 Multivariable analysis and sensitivity analyses

The five prespecified candidate predictors-age, histological subtype, FIGO stage, KELIM and postoperative residual disease-were simultaneously included in the multivariable Cox proportional hazards model. Because postoperative residual disease status was unequivocally available for 92 patients, the primary multivariable analysis included 92 patients with 59 deaths.

Increasing age, advanced FIGO stage, KELIM < 1 and the presence of gross postoperative residual disease were independently associated with poorer OS. Each 10-year increase in age was associated with a 53% increase in the hazard of death (HR, 1.53; 95% CI, 1.18-1.97; P = 0.001). Advanced-stage disease (FIGO III-IV vs. I-II) was associated with a higher mortality risk (HR, 2.56; 95% CI, 1.10-5.96; P = 0.029). KELIM ≥1 was independently associated with a lower mortality risk (HR, 0.52; 95% CI, 0.30-0.91; P = 0.023). Any gross postoperative residual disease was associated with poorer OS compared with R0 (HR, 2.10; 95% CI, 1.15-3.83; P = 0.015). HGSOC histology was not independently associated with OS after multivariable adjustment (HR, 1.79; 95% CI, 0.92-3.49; P = 0.086; **Table 3**).

**Table 3:** Multivariable Cox proportional hazards regression model for overall survival in epithelial ovarian cancer.

Predictor	HR	95% CI	P-value
Age, per 10-year increase	1.53	1.18–1.97	0.001
HGSOC vs. non-HGSOC	1.79	0.92–3.49	0.086
FIGO III–IV vs. I–II	2.56	1.10–5.96	0.029
KELIM ≥1 vs. <1	0.52	0.30–0.91	0.023
Any gross residual disease vs. R0	2.10	1.15–3.83	0.015

The primary multivariable model included 92 patients with complete data for all five prespecified predictors, including 59 deaths. All five predictors were specified *a priori* and entered simultaneously into the model; variable inclusion was not determined by statistical significance in the univariable analyses. HR, hazard ratio; CI, confidence interval; HGSOC, high-grade serous ovarian cancer; FIGO, International Federation of Gynecology and Obstetrics; KELIM, modeled CA125 elimination rate constant K; R0, no gross residual disease.

Extreme-case sensitivity analyses in which the eight patients with indeterminate residual disease status were alternatively classified as R0 or as having gross residual disease yielded materially unchanged estimates for the principal predictors, supporting the robustness of the primary multivariable findings (**Supplementary Table 2**).

### 3.4 Kaplan-Meier survival analysis

**Figure 1:**
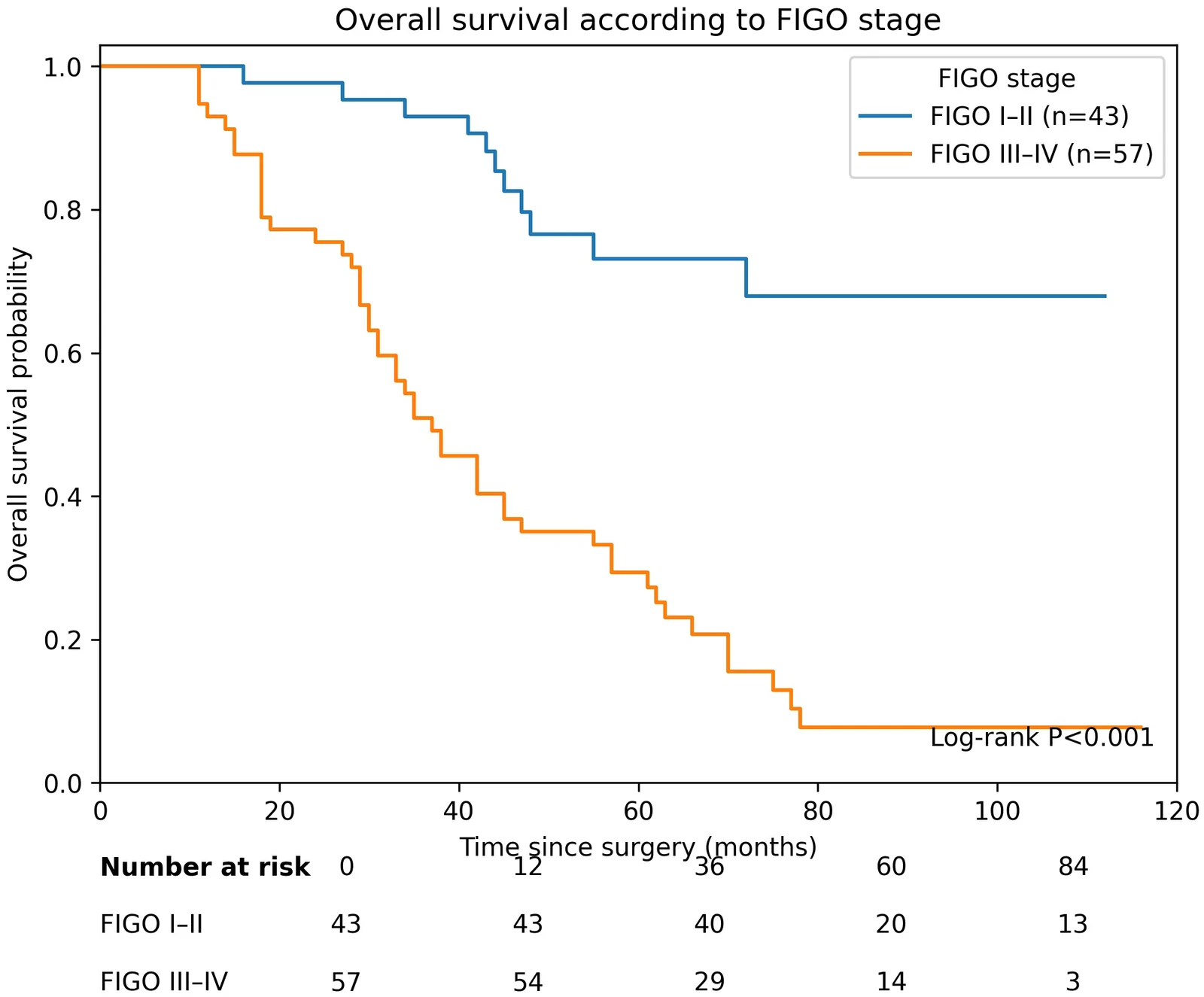
Kaplan–Meier analysis of overall survival according to FIGO stage. Patients with advanced-stage disease (FIGO III–IV) had significantly poorer overall survival than patients with early-stage disease (FIGO I–II) (log-rank P<0.001). FIGO, International Federation of Gynecology and Obstetrics.

**Figure 2:**
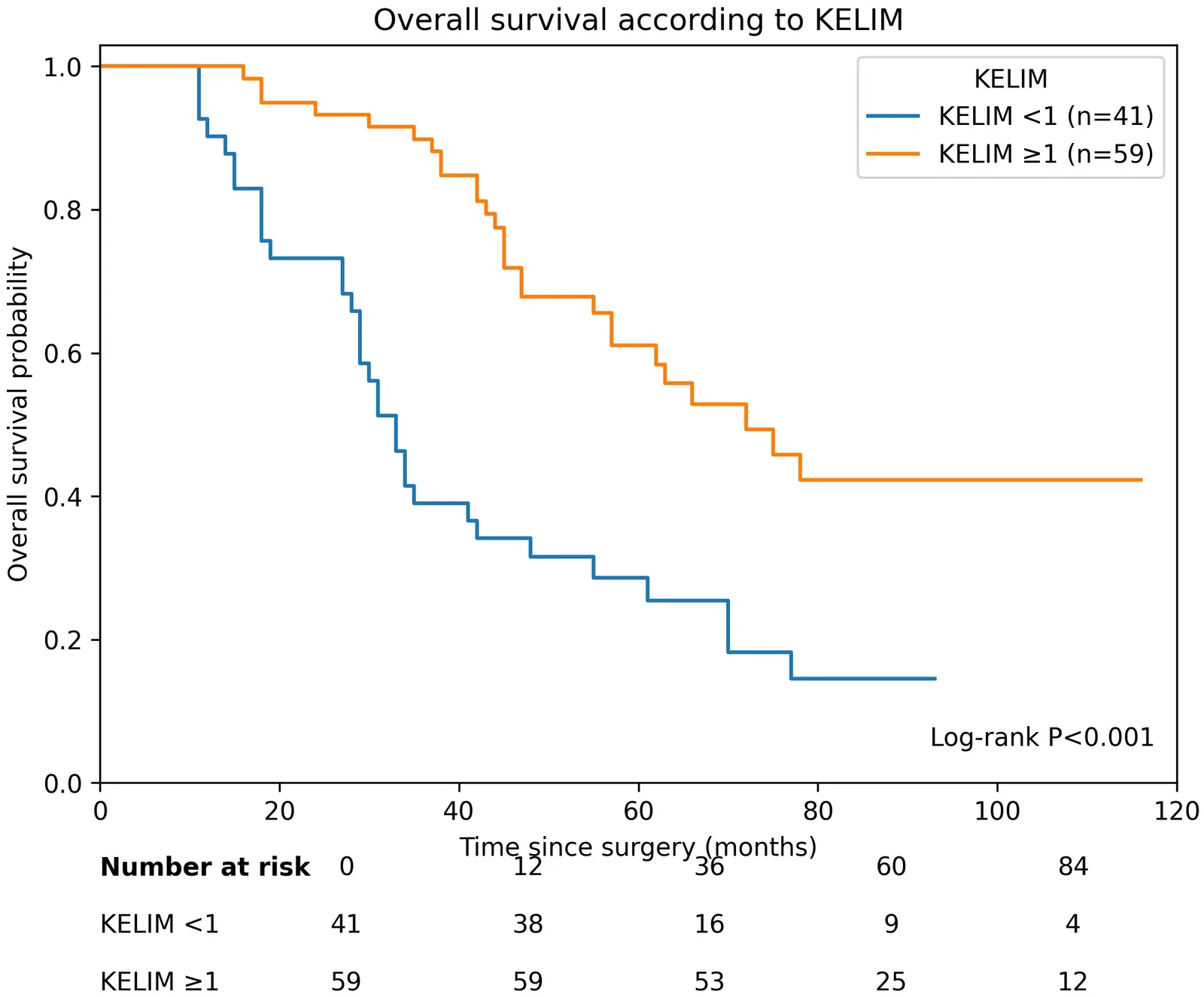
Kaplan–Meier analysis of overall survival according to KELIM. Patients with KELIM ≥1 had significantly better overall survival than those with KELIM <1 (log-rank P<0.001). KELIM, modeled CA125 elimination rate constant K.

**Figure 3:**
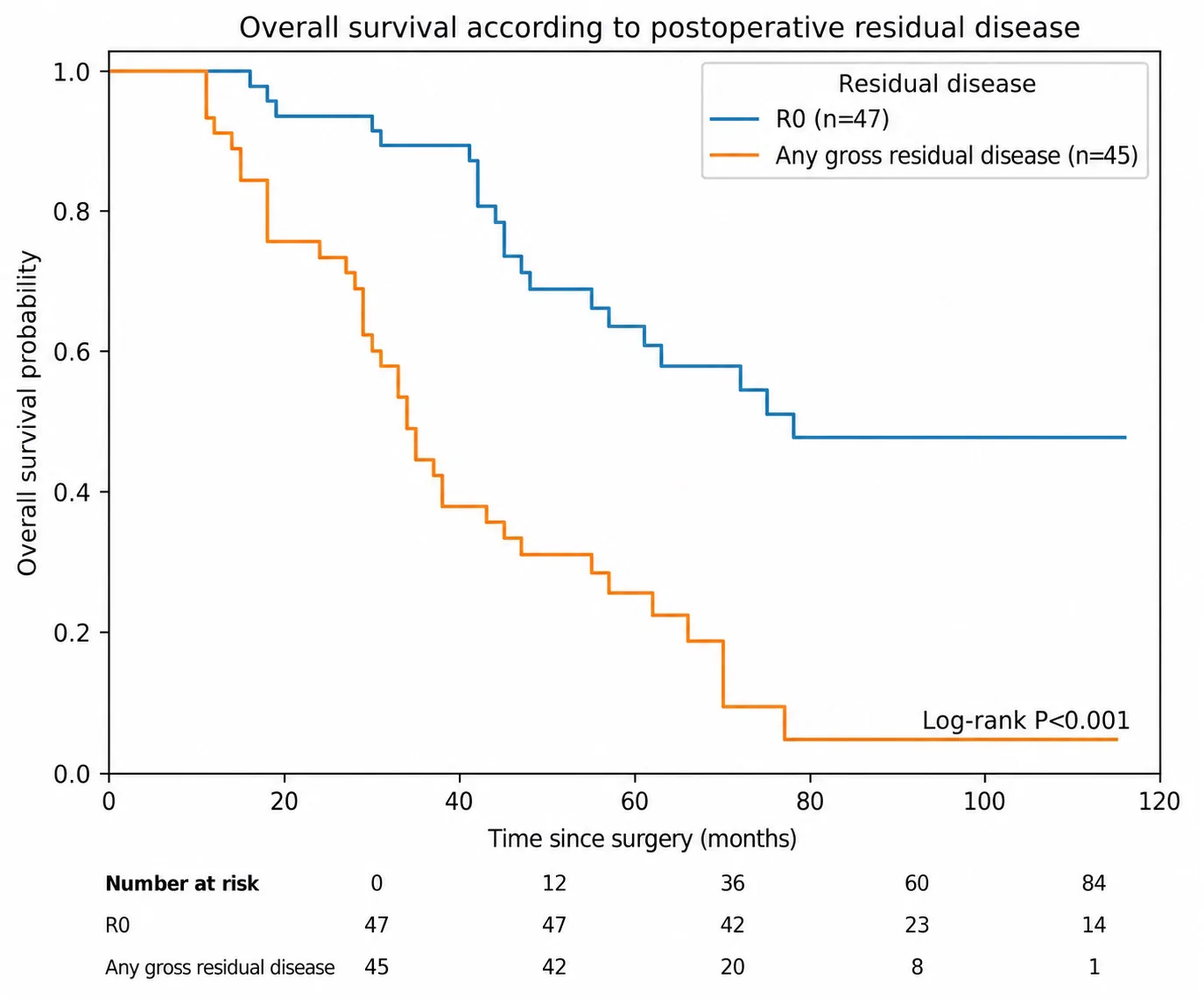
Kaplan–Meier analysis of overall survival according to postoperative residual disease. Among the 92 patients with evaluable postoperative residual disease status, patients who achieved no gross residual disease (R0) had significantly better overall survival than those with any gross residual disease (R1/R2) (log-rank P<0.001). R0, no gross residual disease.

Kaplan-Meier survival analyses demonstrated significant differences in OS according to FIGO stage, KELIM and postoperative residual disease. Patients with early-stage disease (FIGO I-II) had significantly better OS than those with advanced-stage disease (FIGO III-IV; log-rank P<0.001; **Figure 1**). Patients with KELIM ≥1 had significantly better OS than those with KELIM <1 (log-rank P<0.001; **Figure 2**). Among the 92 patients with evaluable postoperative residual disease status, patients who achieved R0 had significantly better OS than those with any gross residual disease (log-rank P<0.001; **Figure 3**).

### 3.5 Nomogram construction and internal validation

**Figure 4:**
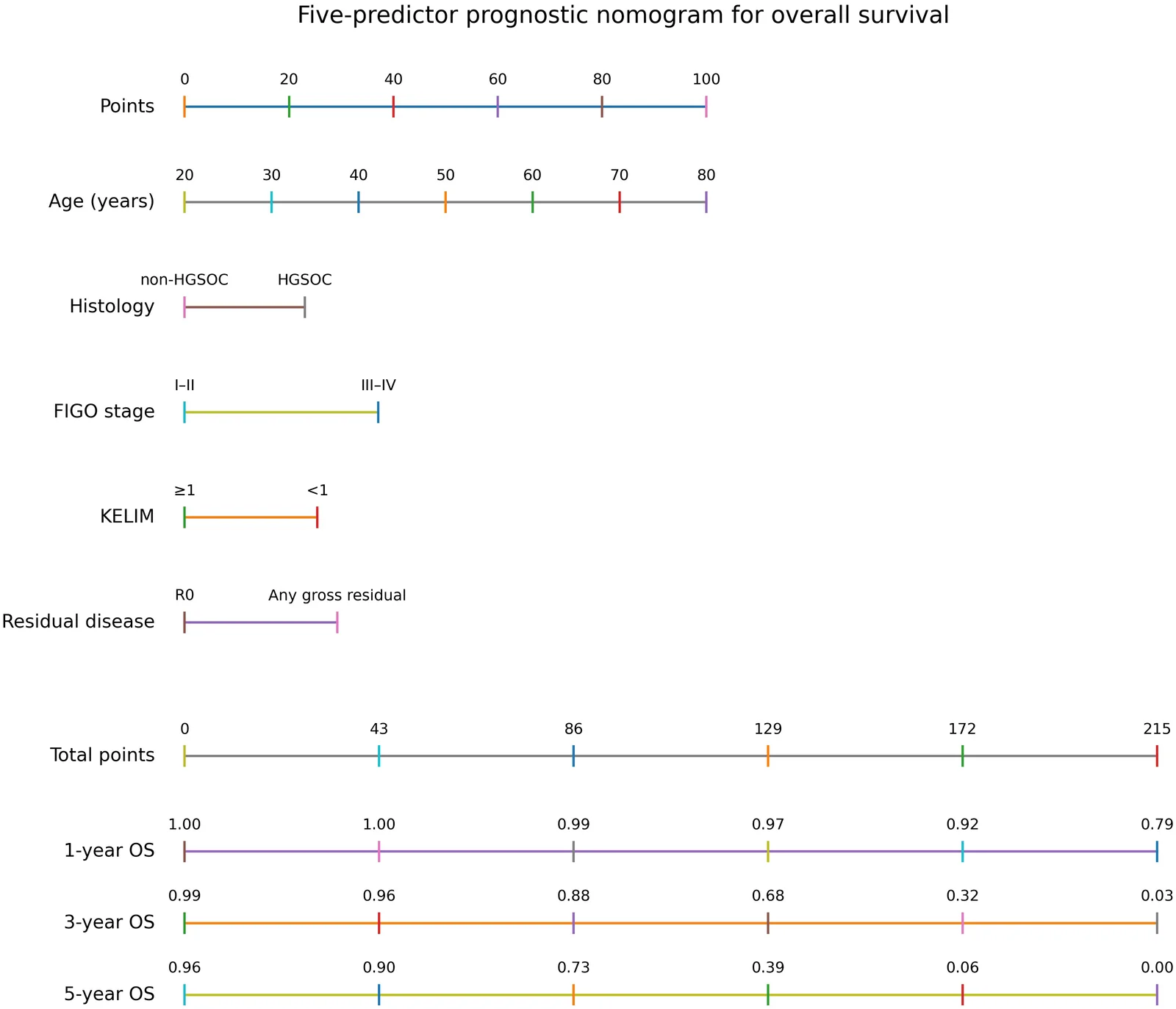
Prognostic nomogram for overall survival in patients with epithelial ovarian cancer. The nomogram was constructed from the full multivariable Cox model containing five prespecified predictors: age, histological subtype, FIGO stage, KELIM and postoperative residual disease. Points assigned to each predictor are summed to obtain a total score, which corresponds to the estimated probabilities of 1-, 3- and 5-year overall survival. The model-development cohort included 92 patients with complete data for all five predictors. FIGO, International Federation of Gynecology and Obstetrics; KELIM, modeled CA125 elimination rate constant K.

A prognostic nomogram was constructed from the full multivariable Cox model containing the five prespecified predictors: age, histological subtype, FIGO stage, KELIM and postoperative residual disease. All five predictors were retained in the model irrespective of their individual statistical significance. The total point score generated by the nomogram was used to estimate individual 1-, 3- and 5-year OS probabilities (**Figure 4**).

**Figure 5:**
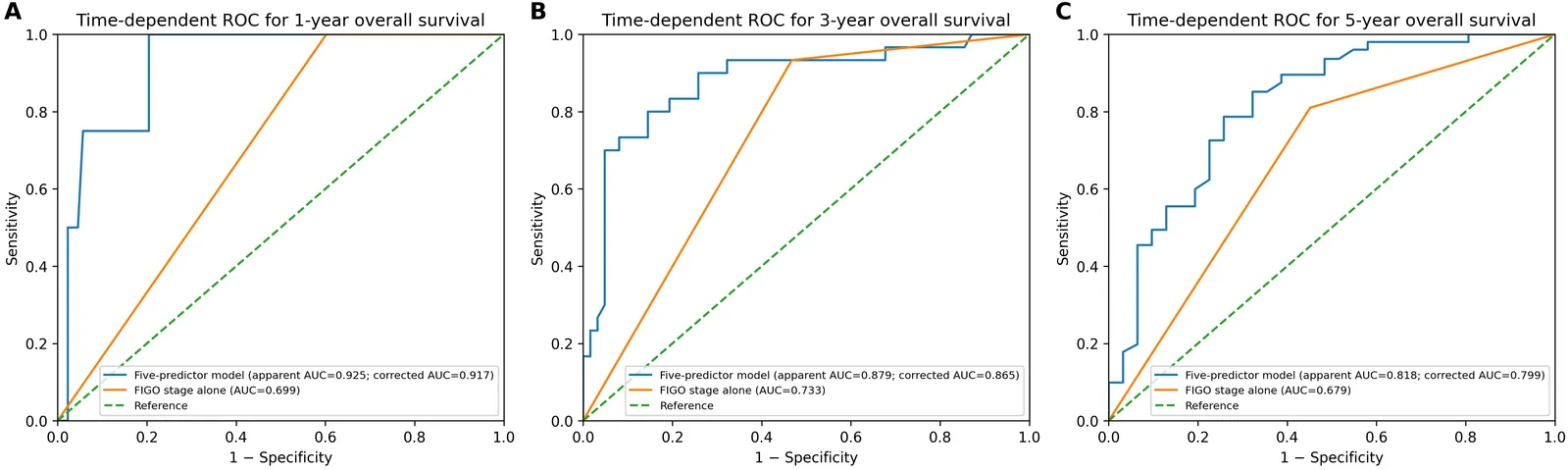
Time-dependent receiver operating characteristic analyses of the five-predictor prognostic model and FIGO stage alone. Time-dependent ROC curves were generated for prediction of **(A)** 1-year, **(B)** 3-year and **(C)** 5-year overall survival. The plotted ROC curves represent apparent model performance. The apparent AUCs of the five-predictor model were 0.925, 0.879 and 0.818 at 1, 3 and 5 years, respectively, and the corresponding optimism-corrected AUCs after 1,000 bootstrap resamples were 0.917, 0.865 and 0.799. The corresponding AUCs for FIGO stage alone were 0.699, 0.733 and 0.679. ROC, receiver operating characteristic; AUC, area under the curve; FIGO, International Federation of Gynecology and Obstetrics.

The apparent C-index of the five-predictor model was 0.795. Internal validation using 1,000 bootstrap resamples yielded a mean optimism of 0.016, resulting in an optimism-corrected C-index of 0.779. The apparent time-dependent AUCs for predicting 1-, 3- and 5-year OS were 0.925, 0.879 and 0.818, respectively. After bootstrap correction, the corresponding AUCs were 0.917, 0.865 and 0.799. These corrected estimates remained higher than the corresponding AUCs for FIGO stage alone (0.699, 0.733 and 0.679, respectively; **Figures 5A–C**).

**Figure 6:**
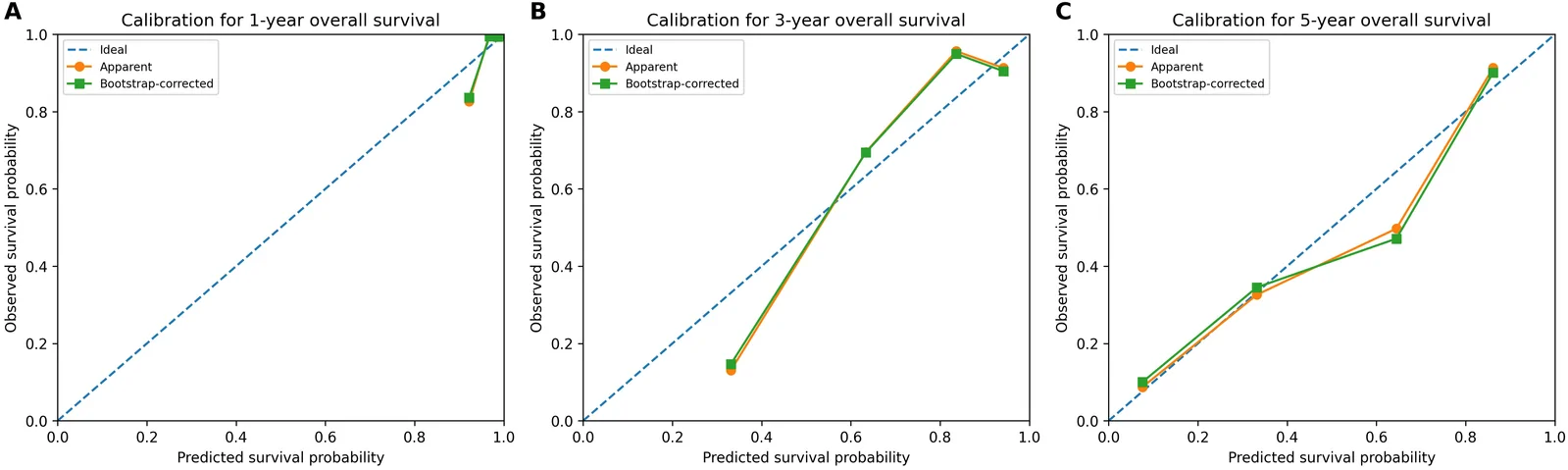
Calibration of the five-predictor prognostic model for overall survival. Calibration plots compare predicted and observed probabilities of **(A)** 1-year, **(B)** 3-year and **(C)** 5-year overall survival. The diagonal line represents perfect agreement between predicted and observed survival. Apparent and optimism-corrected calibration estimates are shown; optimism correction was based on 1,000 bootstrap resamples.

**Figure 7:**
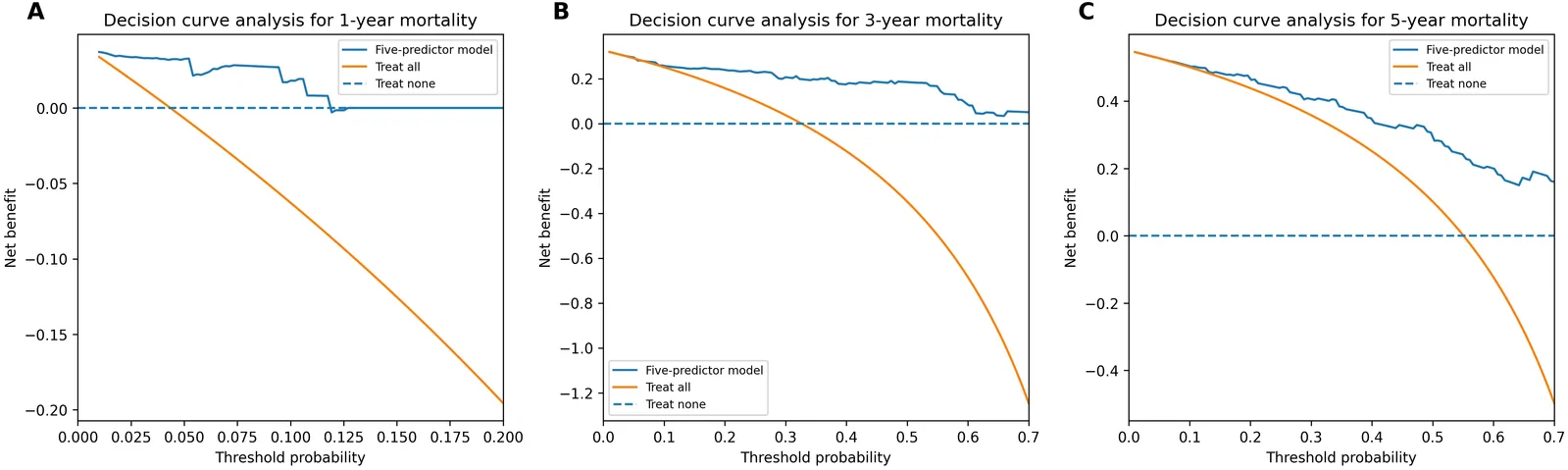
Decision curve analysis of the five-predictor prognostic model. Decision curve analyses were performed to explore the potential clinical net benefit of the model for predicting **(A)** 1-year, **(B)** 3-year and **(C)** 5-year mortality across a range of threshold probabilities. The model is compared with the treat-all and treat-none strategies. DCA, decision curve analysis.

Bootstrap-corrected calibration analyses showed overall reasonable agreement between predicted and observed 1-, 3- and 5-year survival probabilities, although some deviation was observed in selected risk strata (**Figures 6A–C**). Decision curve analysis was additionally performed to explore the potential clinical net benefit of the model across a range of threshold probabilities for 1-, 3- and 5-year mortality (**Figures 7A–C**).

## 4 Discussion

In the present retrospective cohort study, increasing age, advanced FIGO stage, KELIM <1 and gross postoperative residual disease were independently associated with poorer overall survival in patients with epithelial ovarian cancer. By integrating these clinically relevant factors together with histological subtype in a prespecified five-predictor model, we developed a prognostic nomogram combining baseline patient characteristics, tumor histology, anatomical disease extent, surgical outcome and early CA125 kinetics. Following internal validation with 1,000 bootstrap resamples, the model retained acceptable discrimination, with an optimism-corrected C-index of 0.779.

Age remained independently associated with OS after adjustment for the other prespecified clinical variables. Each 10-year increase in age was associated with an approximately 53% increase in the hazard of death. Older patients with EOC may have a greater burden of comorbidities, reduced physiological reserve and lower tolerance of extensive cytoreductive surgery or systemic therapy, which may contribute to inferior long-term outcomes. Importantly, modeling age as a continuous variable avoided the loss of information and potential bias associated with selecting an outcome-dependent cutoff.

FIGO stage remains the foundation of clinical staging, treatment planning and prognostic evaluation in EOC (4). In the present study, patients with FIGO III-IV disease had significantly poorer OS than those with FIGO I-II disease, and advanced stage remained independently associated with mortality after multivariable adjustment. These findings are consistent with previous evidence showing marked survival differences according to disease extent (5). However, FIGO stage primarily reflects anatomical tumor burden at diagnosis and does not fully capture interpatient heterogeneity in tumor biology or treatment response. This limitation supports the incorporation of additional clinical and dynamic response indicators into individualized prognostic assessment.

Postoperative residual disease was also independently associated with OS. Patients who achieved complete macroscopic cytoreduction with no gross residual disease (R0) had significantly better survival than those with any gross residual disease. In the multivariable model, the presence of gross residual disease was associated with an approximately twofold higher hazard of death compared with R0 resection. This finding is consistent with previous clinical and systematic evidence showing that complete macroscopic cytoreduction is a major determinant of survival in ovarian cancer (6, 14). The present results therefore reinforce the contemporary surgical objective of achieving no gross residual disease whenever this can be accomplished safely.

A key finding of the present study was the independent prognostic value of KELIM. Unlike a single CA125 measurement, KELIM reflects the dynamic decline of CA125 during the early phase of platinum-based chemotherapy and provides an indirect measure of tumor chemosensitivity (8). In the present cohort, KELIM ≥1 was independently associated with a lower hazard of death after adjustment for age, histological subtype, FIGO stage and postoperative residual disease. This finding is consistent with previous studies demonstrating associations between favorable KELIM and improved chemotherapy response and survival outcomes, including analyses from the CHIVA study, CALYPSO trial and GOG-0218 study (8–10). Additional analyses from ICON-7 have suggested that KELIM may also help characterize differential benefit from bevacizumab-containing first-line treatment (15), while more recent studies have explored its prognostic relevance in patients receiving PARP inhibitor maintenance therapy (16). Collectively, these observations support KELIM as a clinically relevant dynamic biomarker that provides information on treatment response beyond static anatomical and pathological characteristics.

Exploratory analyses further showed that KELIM was associated with several indicators of disease burden and tumor dissemination, including FIGO stage, postoperative residual disease, preoperative CA125 level, ascites volume, malignant cells in ascites and omental metastasis. By contrast, significant associations were not observed with age or histological subtype in the final dataset. Patients with KELIM <1 more frequently exhibited advanced-stage disease and other adverse disease-related characteristics, suggesting that unfavorable CA125 elimination kinetics may partly reflect greater tumor burden and reduced chemosensitivity. Nevertheless, KELIM remained independently associated with OS after multivariable adjustment, indicating that its prognostic contribution was not fully explained by anatomical disease extent or surgical outcome.

Nomograms provide an intuitive approach to individualized risk prediction by integrating multiple prognostic variables into a single scoring system (17). Previous ovarian cancer nomograms have predominantly relied on baseline clinicopathological factors such as age, stage, histology, tumor markers and treatment-related variables (11, 12). The present model additionally incorporated KELIM as a dynamic indicator of early treatment response. The apparent C-index was 0.795 and decreased modestly to 0.779 after bootstrap correction for optimism, emphasizing the importance of accounting for overoptimistic performance estimates in a relatively small development cohort. Similarly, the optimism-corrected time-dependent AUCs were 0.917, 0.865 and 0.799 for 1-, 3- and 5-year OS, respectively, and remained higher than those for FIGO stage alone (0.699, 0.733 and 0.679, respectively). The high 1-year AUC should nevertheless be interpreted cautiously because only a small number of deaths occurred during the first year of follow-up. Bootstrap-corrected calibration demonstrated overall reasonable agreement between predicted and observed survival probabilities, although some deviation remained in selected risk strata. Decision curve analysis further suggested potential net clinical benefit across selected threshold probabilities; however, these findings require confirmation in independent populations.

From a clinical perspective, the model has the advantage of using variables that are routinely available in clinical practice, including age, histological subtype, FIGO stage, postoperative residual disease and serial CA125 measurements required for KELIM calculation. The model may therefore provide a practical framework for identifying patients at increased risk of poor long-term survival and for refining the intensity of prognostic assessment and follow-up. However, the present study does not establish that treatment should be modified on the basis of KELIM or the nomogram score. Prospective studies are required to determine whether risk-adapted or KELIM-guided treatment strategies improve clinical outcomes.

Several limitations should be acknowledged. First, this was a retrospective single-center study with a relatively small sample size, which may introduce selection bias and limit generalizability. Although model complexity was deliberately restricted to five prespecified predictors, model development and internal validation were based on 92 complete cases with 59 deaths. Secondly, postoperative residual disease status could not be reliably classified in eight patients. These patients were excluded from analyses requiring residual disease information; however, extreme-case sensitivity analyses in which all eight patients were alternatively assigned to R0 or gross residual disease yielded materially unchanged estimates for the principal predictors. Thirdly, internal validation using bootstrap resampling cannot replace external validation in an independent cohort. The small numbers of patients with FIGO stage II and IV disease also precluded stable stage-specific hazard estimates and necessitated grouping stages I-II and III-IV for regression modeling. In addition, only four deaths occurred within the first year, and the 1-year discrimination estimate should therefore be interpreted cautiously. Fourthly, molecular biomarkers such as BRCA mutation and homologous recombination deficiency status were not included in the model, although these factors are increasingly relevant to treatment selection, particularly for PARP inhibitor maintenance therapy (18). In addition, the decision curve analysis, performed according to the framework proposed by Vickers and Elkin (19), should be considered exploratory and requires confirmation in independent cohorts. Finally, KELIM was calculated from available serial CA125 measurements during chemotherapy, and further studies are needed to determine the performance of the model across different histological subtypes, treatment strategies and clinical settings.

In conclusion, the present study developed and internally validated a five-predictor prognostic nomogram incorporating age, histological subtype, FIGO stage, postoperative residual disease and KELIM for long-term survival prediction in patients with epithelial ovarian cancer. Increasing age, advanced FIGO stage, KELIM <1 and gross postoperative residual disease were independently associated with poorer overall survival. After correction for optimism using bootstrap resampling, the model retained acceptable discrimination and showed improved predictive performance compared with FIGO stage alone. These findings support the potential value of combining dynamic CA125 kinetics with established clinical, pathological and surgical factors for individualized prognostic assessment. External validation in larger, independent and multicenter cohorts is required before clinical implementation.

## Data Availability

The raw data supporting the conclusions of this article will be made available by the authors, without undue reservation.

## Supplementary material

Supplementary Table 1 Association of KELIM with selected clinical and clinicopathological characteristics. Continuous variables were compared using the Mann–Whitney U test and categorical variables using the χ² test or Fisher’s exact test, as appropriate. *Postoperative residual disease status was evaluable in 39 patients with KELIM <1 and 53 patients with KELIM ≥1. †An exact numerical preoperative CA125 value was available in 40 patients with KELIM <1 and 59 patients with KELIM ≥1. ‡An exact ascites volume was available in 41 patients with KELIM <1 and 58 patients with KELIM ≥1. BMI, body mass index; HGSOC, high-grade serous ovarian cancer; FIGO, International Federation of Gynecology and Obstetrics; KELIM, modeled CA125 elimination rate constant K; R0, no gross residual disease.

Supplementary Table 2 Sensitivity analyses for alternative classifications of patients with indeterminate postoperative residual disease status. The eight patients with indeterminate postoperative residual disease status were excluded from the primary complete-case analysis. Extreme-case sensitivity analyses were performed by alternatively assigning all eight patients to R0 or to the gross residual disease group. These scenarios were used solely to assess robustness and should not be interpreted as imputed residual disease classifications. HR, hazard ratio; CI, confidence interval; HGSOC, high-grade serous ovarian cancer; FIGO, International Federation of Gynecology and Obstetrics; KELIM, modeled CA125 elimination rate constant K; R0, no gross residual disease.
