# Supplementary material for: Development and internal validation of a KELIM-integrated prognostic nomogram for long-term survival prediction in epithelial ovarian cancer
Source: Front Oncol. 2026 Aug 31;16:1934729. doi: 10.3389/fonc.2026.1934729 (PMC13572248; doi:10.3389/fonc.2026.1934729)
Supplement: Supplementary file 1 [file Table1.docx]

Supplementary Material

**Development and internal validation of a KELIM-integrated prognostic nomogram for long-term survival prediction in epithelial ovarian cancer**

Manuscript ID: 1934729

**Supplementary Table 1. Association of KELIM with selected clinical and clinicopathological characteristics**

| **Variable** | **KELIM <1 (n=41)** | **KELIM ≥1 (n=59)** | **P-value** |
| --- | --- | --- | --- |
| Age, years, median (IQR) | 50.0 (44.0–61.0) | 52.0 (46.5–57.5) | 0.763 |
| BMI, kg/m², median (IQR) | 23.6 (21.6–26.6) | 24.1 (22.0–26.7) | 0.563 |
| **Histological subtype, n (%)** |  |  | 0.154 |
| HGSOC | 26 (63.4) | 28 (47.5) |  |
| Non-HGSOC | 15 (36.6) | 31 (52.5) |  |
| **FIGO stage, n (%)** |  |  | <0.001 |
| I–II | 8 (19.5) | 35 (59.3) |  |
| III–IV | 33 (80.5) | 24 (40.7) |  |
| **Postoperative residual disease, n (%)*** |  |  | <0.001 |
| R0 | 11 (28.2) | 36 (67.9) |  |
| Any gross residual disease | 28 (71.8) | 17 (32.1) |  |
| Preoperative CA125, U/ml, median (IQR)† | 1395.0 (504.5–2750.5) | 340.6 (50.6–897.5) | <0.001 |
| Ascites volume, ml, median (IQR)‡ | 500 (50–2700) | 100 (0–875) | 0.016 |
| **Malignant cells in ascites, n (%)** |  |  | 0.008 |
| Absent | 11 (26.8) | 32 (54.2) |  |
| Present | 30 (73.2) | 27 (45.8) |  |
| **Omental metastasis, n (%)** |  |  | 0.001 |
| Absent | 14 (34.1) | 40 (67.8) |  |
| Present | 27 (65.9) | 19 (32.2) |  |
| **Liver metastasis, n (%)** |  |  | 1.000 |
| Absent | 40 (97.6) | 57 (96.6) |  |
| Present | 1 (2.4) | 2 (3.4) |  |
| Interval from surgery to chemotherapy initiation, days, median (IQR) | 11 (10–14) | 11 (10–14.5) | 0.732 |
| Number of chemotherapy cycles, median (IQR) | 7 (6–8) | 6 (6–8) | 0.370 |
| **Chemotherapy cycles, n (%)** |  |  | 0.226 |
| ≤6 | 19 (46.3) | 35 (59.3) |  |
| >6 | 22 (53.7) | 24 (40.7) |  |

Continuous variables were compared using the Mann–Whitney U test and categorical variables using the χ² test or Fisher's exact test, as appropriate. *Postoperative residual disease status was evaluable in 39 patients with KELIM <1 and 53 patients with KELIM ≥1. †An exact numerical preoperative CA125 value was available in 40 patients with KELIM <1 and 59 patients with KELIM ≥1. ‡An exact ascites volume was available in 41 patients with KELIM <1 and 58 patients with KELIM ≥1. BMI, body mass index; HGSOC, high-grade serous ovarian cancer; FIGO, International Federation of Gynecology and Obstetrics; KELIM, modeled CA125 elimination rate constant K; R0, no gross residual disease.
