# Supplementary material for: Development and internal validation of a KELIM-integrated prognostic nomogram for long-term survival prediction in epithelial ovarian cancer
Source: Front Oncol. 2026 Aug 31;16:1934729. doi: 10.3389/fonc.2026.1934729 (PMC13572248; doi:10.3389/fonc.2026.1934729)
Supplement: Supplementary file 2 [file Table2.docx]

**Supplementary Table 2. Sensitivity analyses for alternative classifications of patients with indeterminate postoperative residual disease status**

| **Analysis** | **Predictor** | **HR** | **95% CI** | **P-value** |
| --- | --- | --- | --- | --- |
| **Primary complete-case analysis (n=92; 59 deaths)** | Age, per 10-year increase | 1.53 | 1.18–1.97 | 0.001 |
|  | HGSOC vs. non-HGSOC | 1.79 | 0.92–3.49 | 0.086 |
|  | FIGO III–IV vs. I–II | 2.56 | 1.10–5.96 | 0.029 |
|  | KELIM ≥1 vs. <1 | 0.52 | 0.30–0.91 | 0.023 |
|  | Any gross residual disease vs. R0 | 2.10 | 1.15–3.83 | 0.015 |
| **Extreme-case analysis 1: all 8 indeterminate patients classified as R0 (n=100)** | Age, per 10-year increase | 1.47 | 1.15–1.88 | 0.002 |
|  | HGSOC vs. non-HGSOC | 1.81 | 0.94–3.50 | 0.076 |
|  | FIGO III–IV vs. I–II | 2.47 | 1.07–5.69 | 0.033 |
|  | KELIM ≥1 vs. <1 | 0.49 | 0.28–0.84 | 0.010 |
|  | Any gross residual disease vs. R0 | 2.07 | 1.14–3.77 | 0.017 |
| **Extreme-case analysis 2: all 8 indeterminate patients classified as gross residual disease (n=100)** | Age, per 10-year increase | 1.47 | 1.16–1.88 | 0.002 |
|  | HGSOC vs. non-HGSOC | 1.88 | 0.96–3.67 | 0.065 |
|  | FIGO III–IV vs. I–II | 2.70 | 1.19–6.13 | 0.017 |
|  | KELIM ≥1 vs. <1 | 0.48 | 0.28–0.83 | 0.008 |
|  | Any gross residual disease vs. R0 | 1.96 | 1.10–3.47 | 0.022 |

The eight patients with indeterminate postoperative residual disease status were excluded from the primary complete-case analysis. Extreme-case sensitivity analyses were performed by alternatively assigning all eight patients to R0 or to the gross residual disease group. These scenarios were used solely to assess robustness and should not be interpreted as imputed residual disease classifications. HR, hazard ratio; CI, confidence interval; HGSOC, high-grade serous ovarian cancer; FIGO, International Federation of Gynecology and Obstetrics; KELIM, modeled CA125 elimination rate constant K; R0, no gross residual disease.
